# Supplementary material for: Decapod-inspired pigment modulation for active building facades
Source: Nat Commun. 2022 Jul 15;13:4120. doi: 10.1038/s41467-022-31527-6 (PMC9287369; doi:10.1038/s41467-022-31527-6)
Supplement: Supplementary file 1 — Supplementary Information [file 41467_2022_31527_MOESM1_ESM.pdf]

Supplementary Information for

Decapod-inspired pigment modulation for active building facades

Raphael Kay*, Charlie Katrycz, Kevin Nitièma, J. Alstan Jakubiec, Benjamin D. Hatton*

*Correspondence to: raphael.kay@mail.utoronto.ca, benjamin.hatton@utoronto.ca

Supplementary Discussion

Details on the assumption that interfacial velocity of pigment fluid is linearly proportional to injection flow rate

An expanding thin disk of an incompressible fluid of height b and radius r adds the following angular differential volume element:

$$dVol = 2\pi rbdr$$

The flux-interfacial velocity relationship can be easily established,

$$\frac{dVol}{dt} = 2\pi rb\frac{dr}{dt}$$

Using the notation,

$$q = \frac{dVol}{dt}, V = \frac{dr}{dt}$$

We get,

$$q = 2\pi rbV$$

$$V = \frac{q}{2\pi rb}$$

So, we expect that the velocity of the interface in this case is linear with flux, and inversely proportional to the radius. In other words, the velocity of the interface is equal to the volume flux divided by the area of the interface. However, we note that when the interface expands non-stably in fingering protrusions, the velocity of fingertips is increased (up to twice the value derived above as area fraction approaches 50%) due to the concentration of steep pressure gradients at growth tips, while the velocity of the interface between fingertips decreases to zero. The derivation for V above represents the velocity averaged over the interface as it expands non-stably.

Supplementary Note 1

To estimate the performance capacity of our fluid layer across seasonally-dependent temperatures, we calculated the range in injection velocity required to compensate for the change in relative viscosity induced by the maximum and minimum fluctuations in fluid temperature. Our active fluid layer was implemented on the interior of the double-glazed control window described in our manuscript (U-value of 1.81 W/m^2K, visible transmittance of 0.81, and SHGC of 0.71), and the temperature of the fluid layer across the year was calculated in EnergyPlus, using the conduction finite difference solution algorithm[1,2] (Supplementary Figure 14). The maximum fluid temperature was 44.3 °C on August 13th, while the minimum fluid temperature was 8.9 °C on February 23rd. We assumed the fluidic pigment to be a water-glycerol solution (0.8/0.2 by volume) with a freezing point of approximately -10 °C [3], and we calculated pigment fluid viscosities corresponding to

minimum (8.9 °C), maximum (44.3 °C), and laboratory (20 °C) temperatures using the tool developed in [4], from the relationships described in [5,6] (Supplementary Table 1). Using known temperature-dependent viscosities for the host mineral oil[7], we then calculated the viscosity difference to be expected at each relevant fluid temperature within our fluid layer. Then, using the relationships introduced in Equation (2-3), we estimated the required increase or decrease to injection velocity in order to achieve the same characteristic wavelength as for a laboratory temperature condition with the same fluids. From Supplementary Table 1, we can observe that the estimated change in injection velocity to compensate for minimum and maximum temperature deviations from laboratory conditions is within an order of magnitude: at the coldest modelled annual temperature, the injection velocity would need to be lowered to 43% of its laboratory value, while, at the hottest modelled annual temperature, the injection velocity would need to be raised to 415% of its laboratory value. These changes to injection velocity are well within the functional range of our peristaltic pumps, and we imagine the appropriate temperature-dependent performance could be easily integrated within a digital control algorithm.

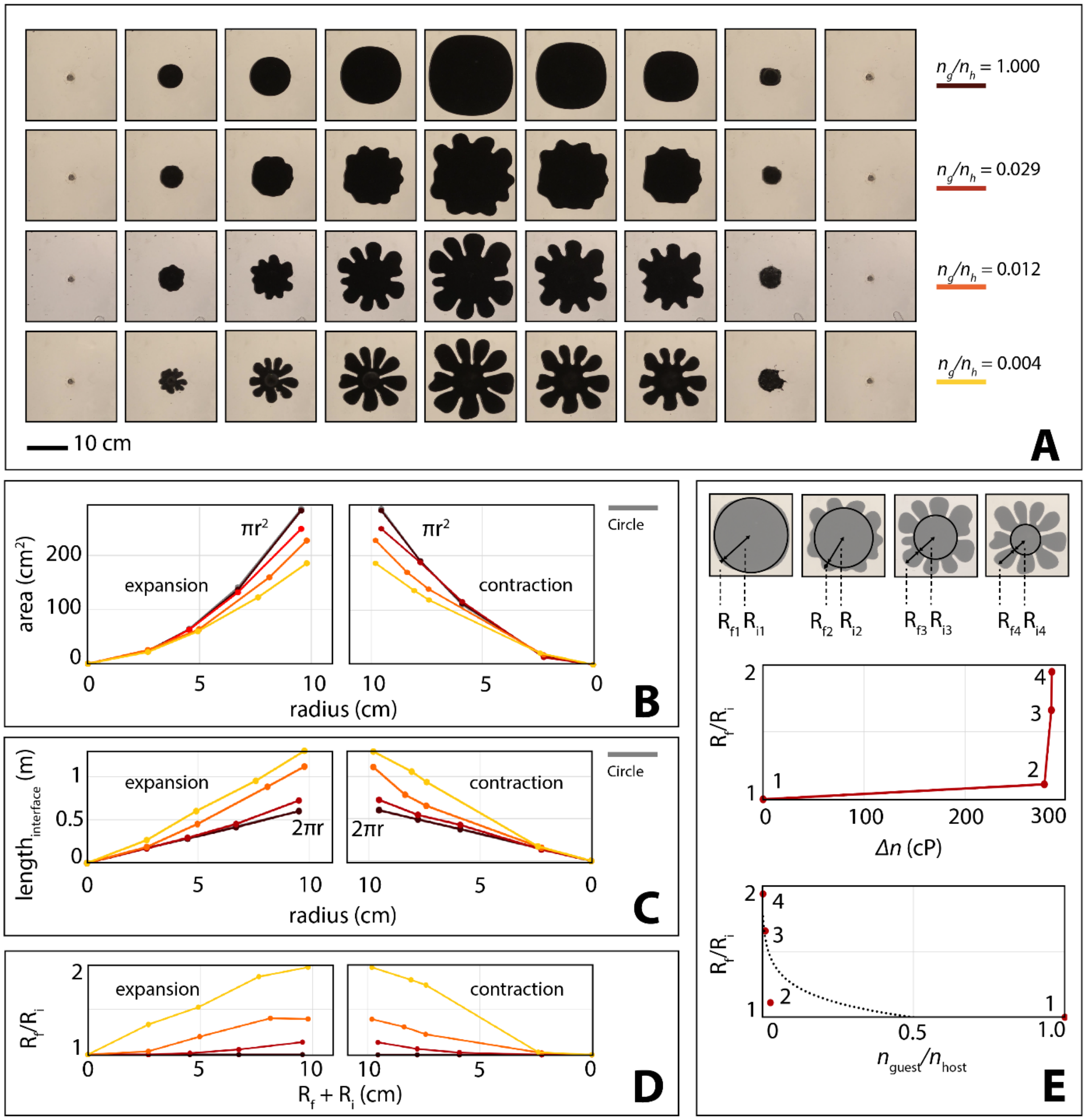

Supplementary Figure 1.

Control over geometrical characteristics of pigment dispersal through tuning of ratio between viscosity of guest fluid (n_g) and viscosity of host fluid (n_h). (a) Four individual pigment injection and retraction sequences, from left to right, over time, with varied ratio between guest and host fluid viscosities. (b) Area of pigment formation as function of pattern radius through expansion and contraction sequence. (c) Interfacial perimeter of pigment formation as function of pattern radius through expansion and contraction sequence. (d) Ratio of circular radii corresponding to inner (R_i) and fingering (R_f) pattern regions as function of total pattern radius. (e) Ratio of circular radii corresponding to inner (R_i) and fingering (R_f) pattern regions as function of viscosity difference and ratio.

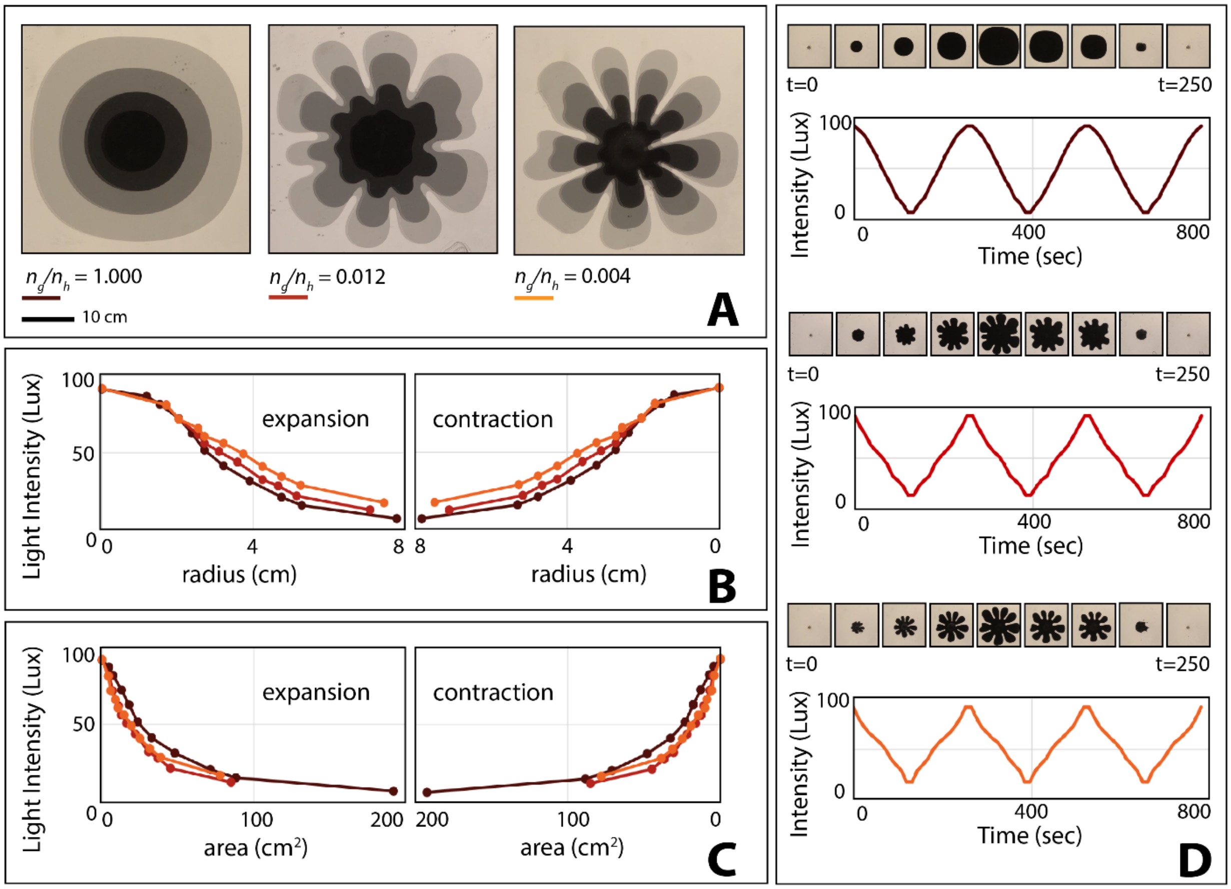

Supplementary Figure 2.

Indirect effect of viscosity difference on light transmission. (a) Overlaid time-series images for three independent pigment injection sequences, where viscosity difference (and ratio) is varied. (b) Relative transmitted light intensity through the Hele-Shaw cell as function of pattern radius. (c) Relative transmitted light intensity as function of pattern area. (d) Light intensity as function of time across three repeated pattern injection and retraction sequences.

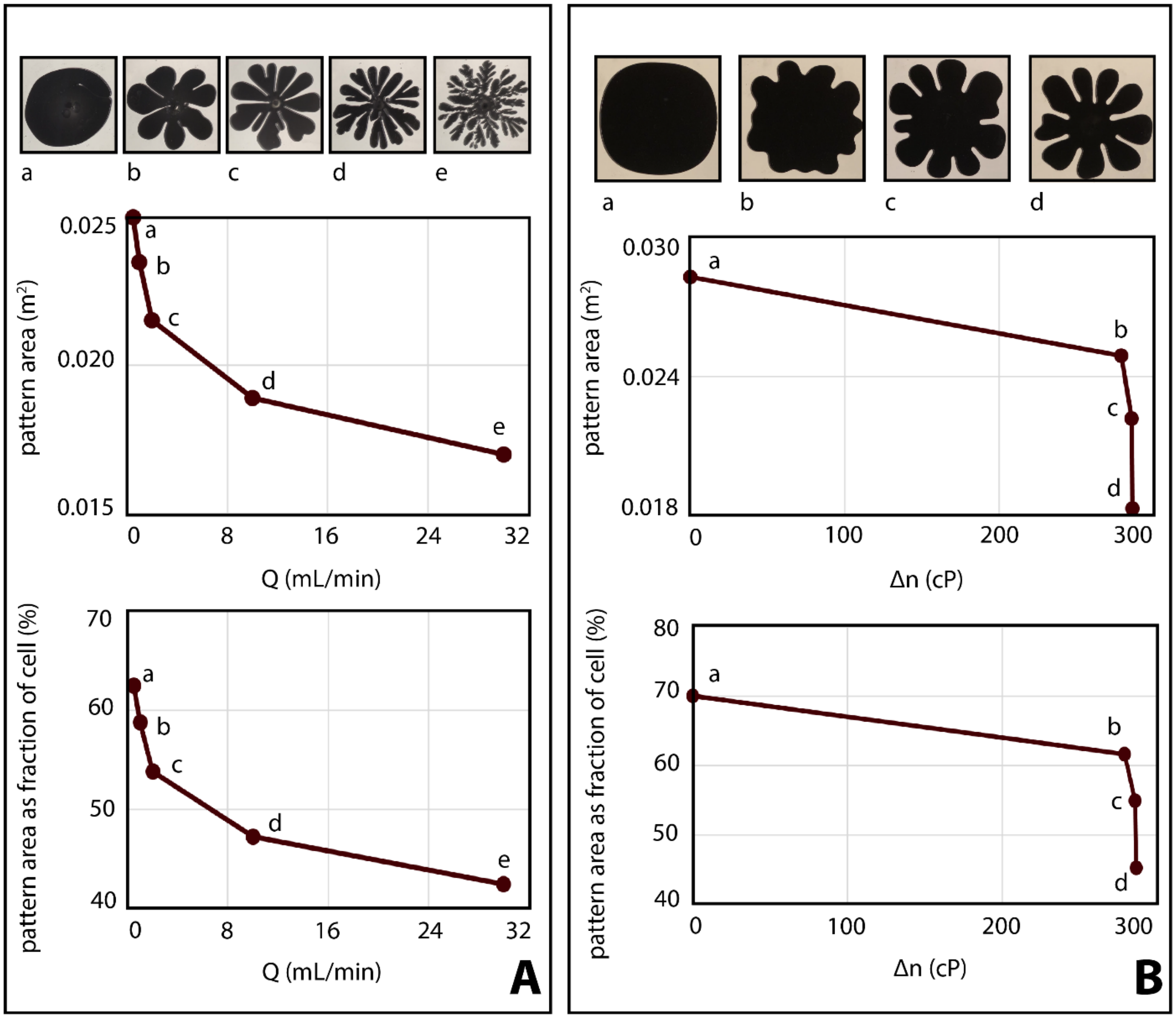

Supplementary Figure 3.

Tuning of pattern area as function of flow rate (a) and viscosity difference (b) for patterns of a comparable radius within a Hele-Shaw cell.

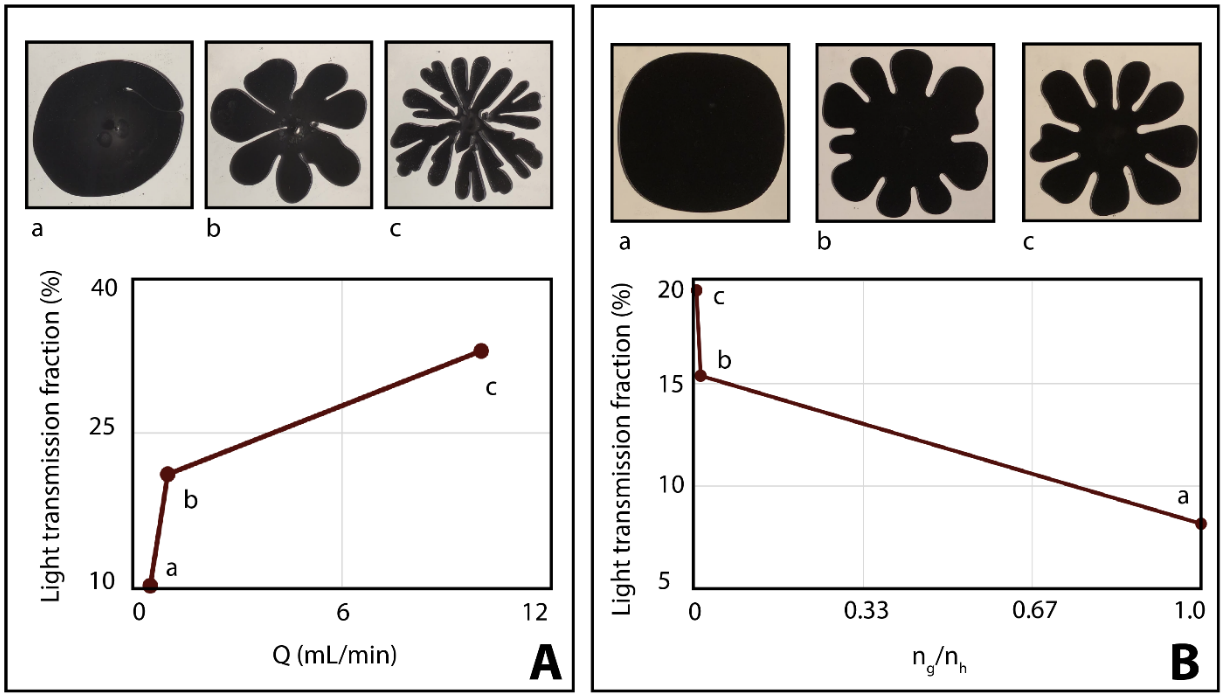

Supplementary Figure 4.

Tuning of light transmission through Hele-Shaw cell for different pattern morphologies as a function of flow rate (a) and viscosity ratio (b). All patterns have an equivalent maximum radius.

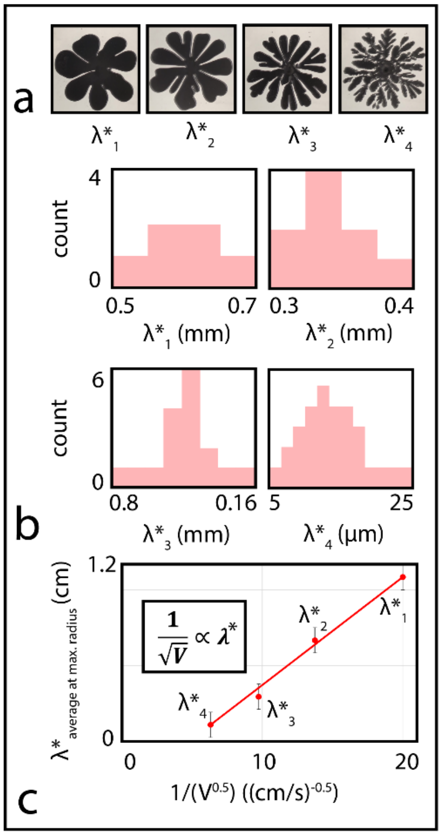

Supplementary Figure 5.

Observed pattern morphology is consistent with equations (2-3), where the characteristic wavelength is proportional to the inverse of the square of V, the observed interfacial velocity (c). Here, the characteristic wavelength (λ^*) is taken as the average wavelength of fingers observed in (a).

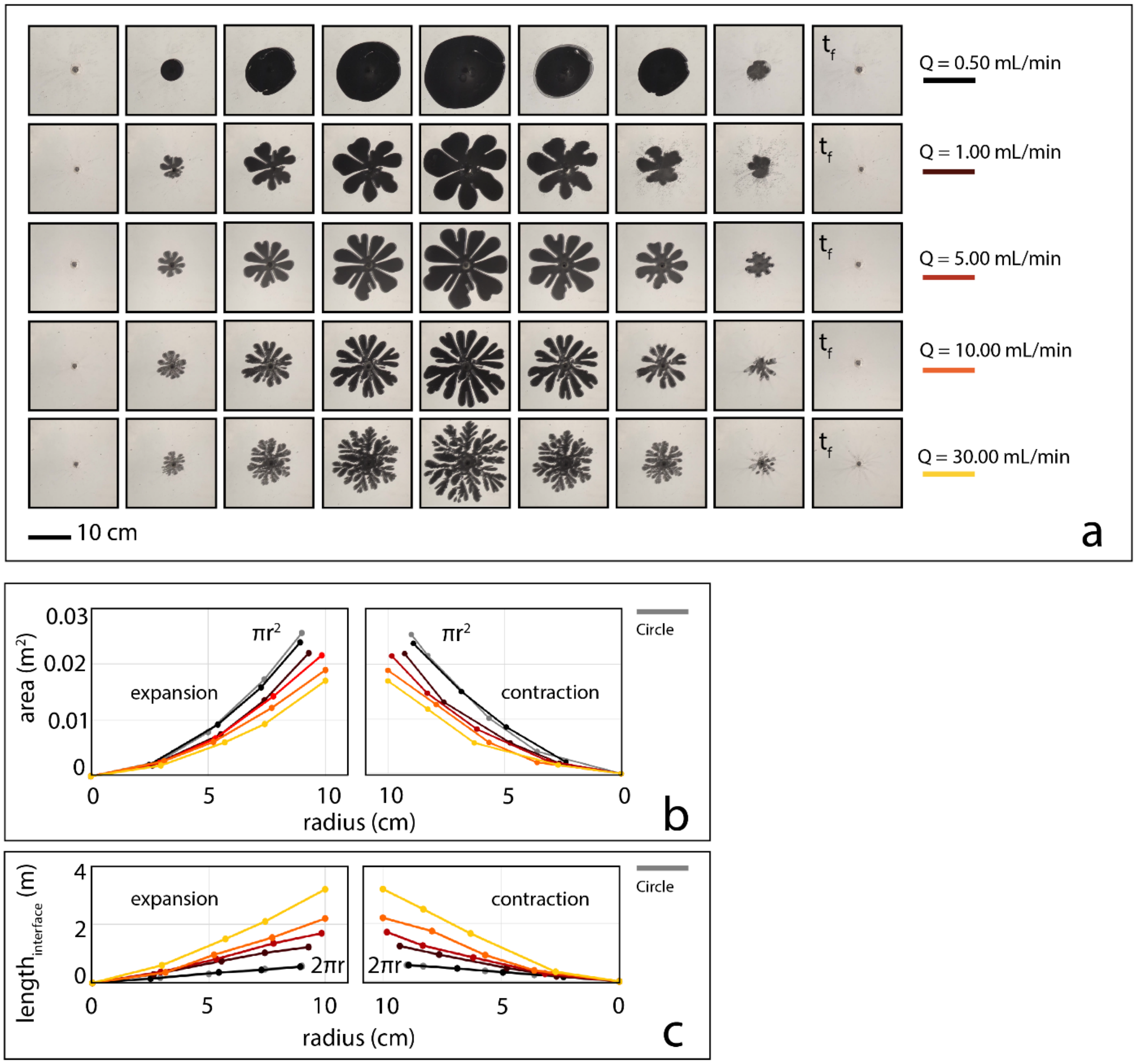

Supplementary Figure 6.

Control over geometrical characteristics of pigment dispersal through tuning of flow rate. (a) Five individual pigment injection and retraction sequences, from left to right, over time, with varied flow rates. (b) Area of pigment formation as function of pattern radius through expansion and contraction sequence. (c) Interfacial perimeter of pigment formation as function of pattern radius through expansion and contraction sequence.

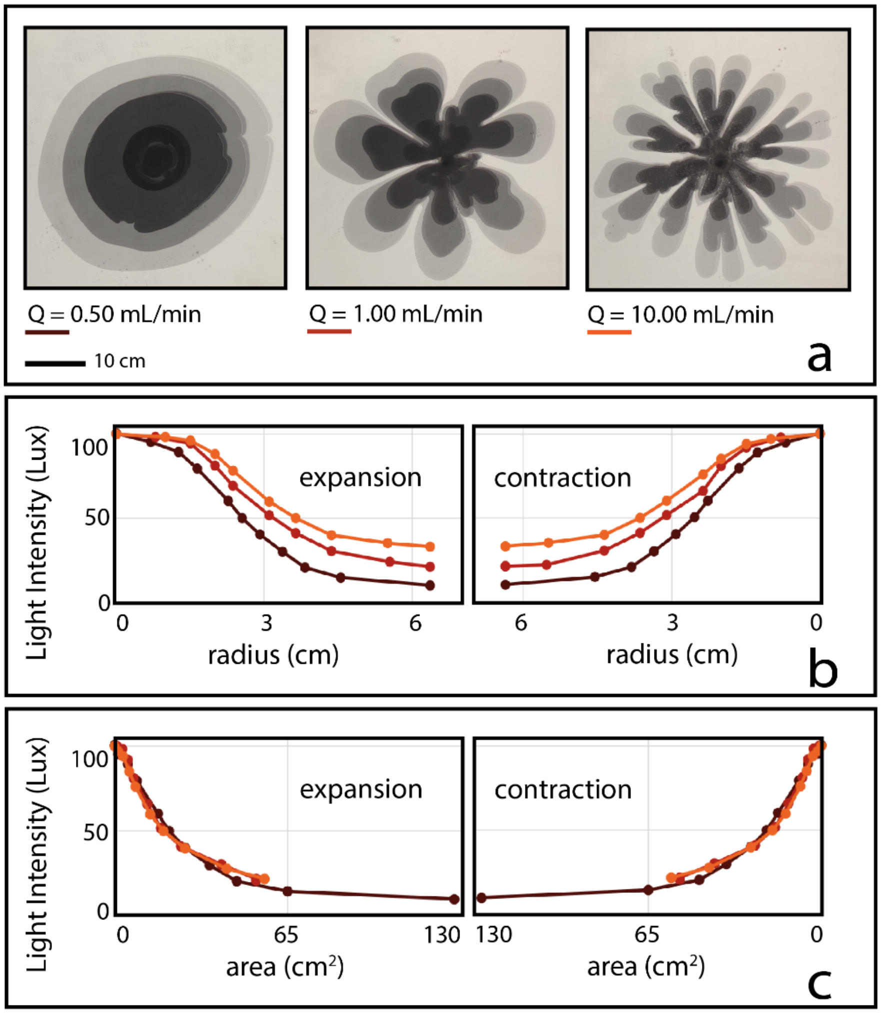

Supplementary Figure 7.

Indirect effect of flow rate on light transmission. (a) Overlaid time-series images for three independent pigment injection sequences, where flow rate is varied. (b) Relative transmitted light intensity through Hele-Shaw cell as function of pattern radius. (c) Relative transmitted light intensity as function of pattern area. Light transmission is dependent only on pattern area.

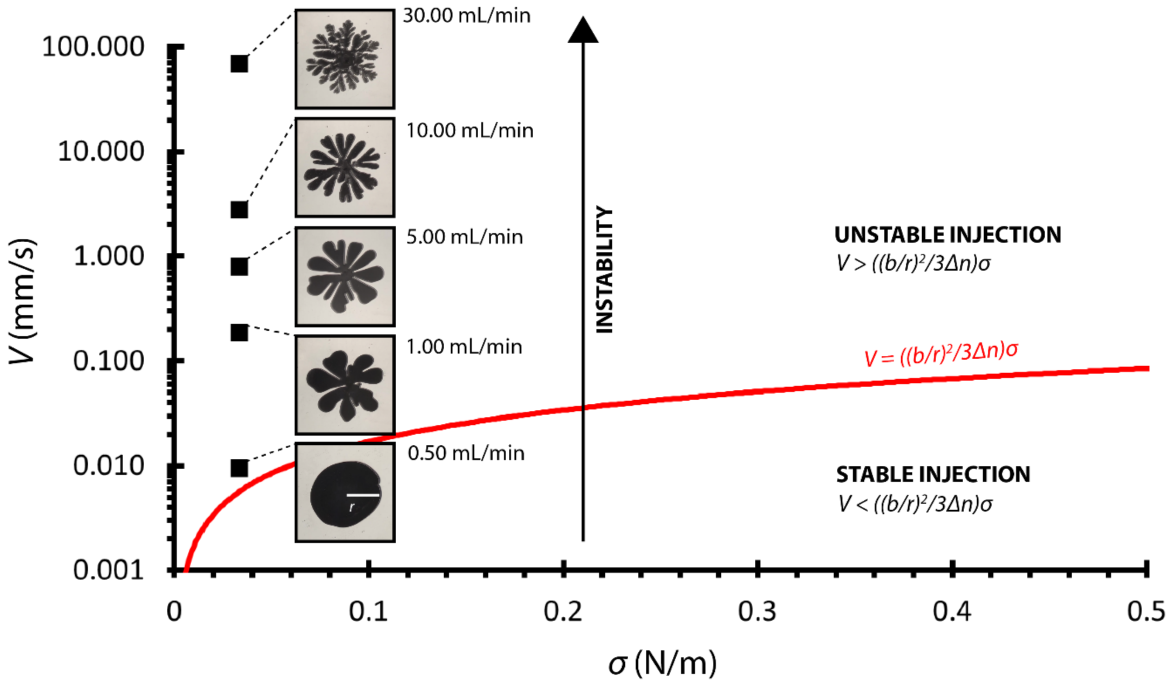

Supplementary Figure 8.

Binary phase space differentiating between stable and unstable pigment injections within a Hele-Shaw cell. One can observe the decrease in characteristic finger width λ^* as the value of V increases, further away from the region of stability. At higher values of σ, greater velocities are needed to support unstable branched growth.

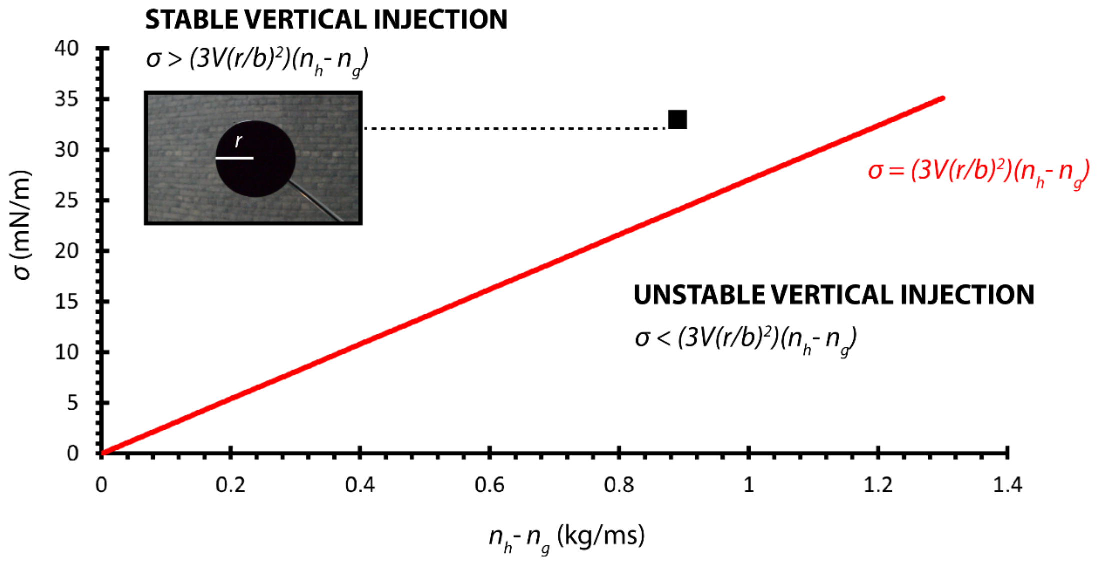

Supplementary Figure 9.

Binary phase space for stable non-branching and unstable branching pigment injection within a vertical Hele-Shaw cell.

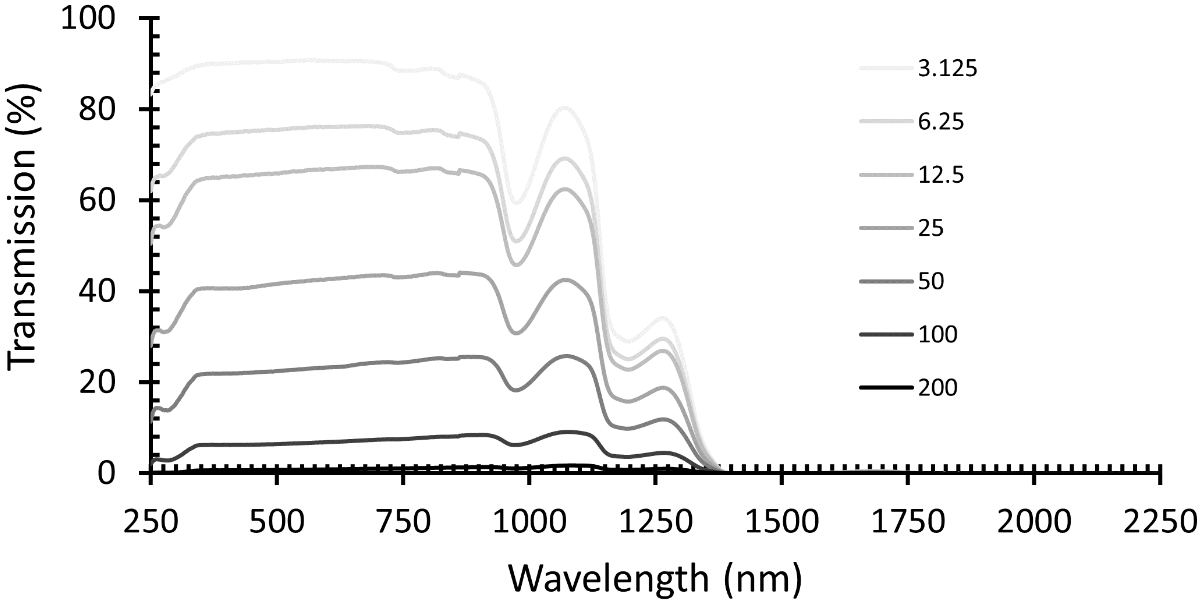

Supplementary Figure 10.

Optical transmission for various aqueous suspensions of carbon black, for a 4-mm-thick fluidic optical path length. Values describe concentration of mg carbon per 50 mL of H_2O.

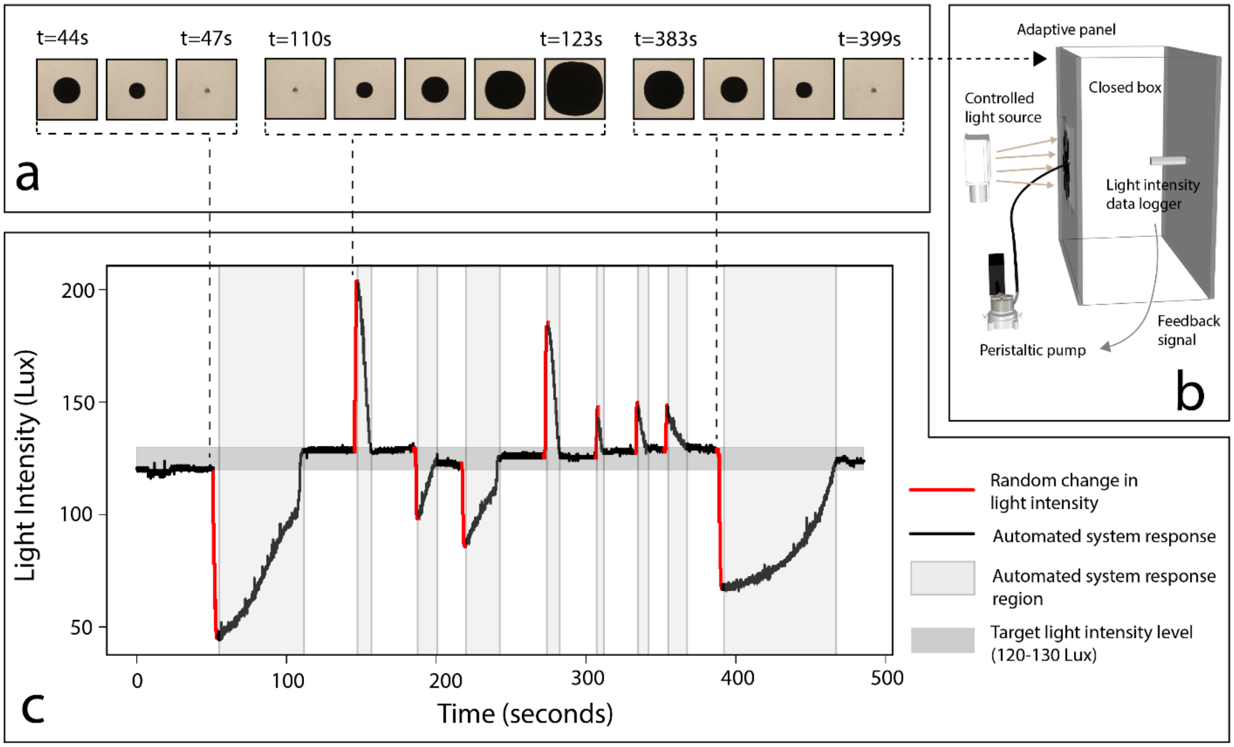

Supplementary Figure 11.

Demonstration of negative optical feedback system. (a) Examples (not directly drawn from this experiment) to illustrate pigment fluid response. (b) Schematic of experiment. (c) Measured interior light intensity, where red curves represent manual interventions to increase or decrease light intensity, and black curves represent digital optical response to change in measured light intensity.

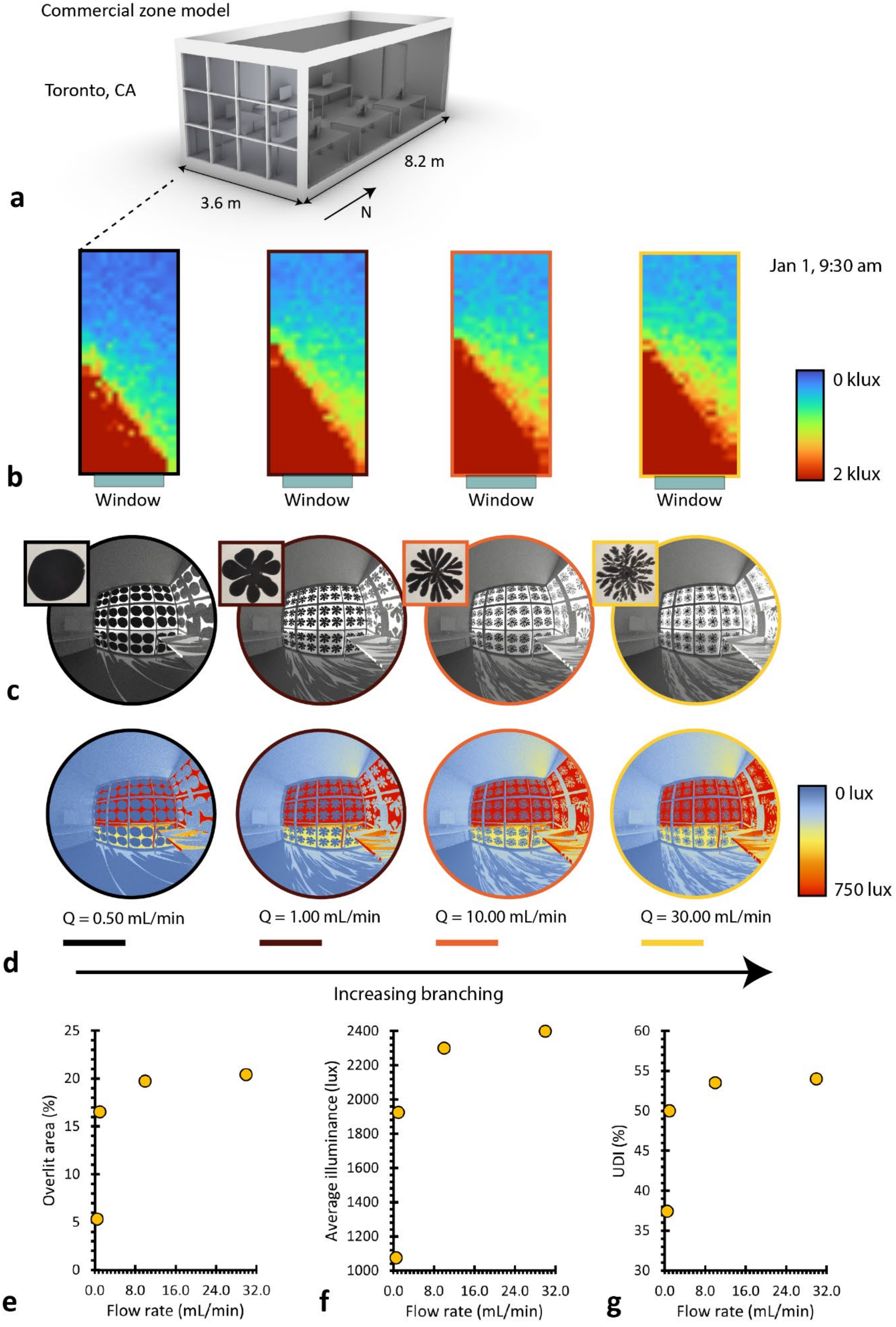

Commercial zone model
Toronto, CA
8.2 m
3.6 m
N
a
Jan 1, 9:30 am
0 klux
2 klux
Window
Window
Window
Window
b
c
0 lux
750 lux
Q = 0.50 mL/min
Q = 1.00 mL/min
Q = 10.00 mL/min
Q = 30.00 mL/min
d
Increasing branching
Overlit area (%)
Average illuminance (lux)
UDI (%)
Flow rate (mL/min)
Flow rate (mL/min)
Flow rate (mL/min)
e
f
g

Supplementary Figure 12.

Differences in indoor illumination for maximum pigment injections at different flow rates (different viscous fingering patterns). (a) Model used for daylight simulation. (b) Illuminance distribution across space (sensors spaced every 0.2 m, and modelling plane taken at 0.762 m). (c-d) Renderings within room taken on Jan 1 at 9:30 am, where (c) shows RGB render and (d) shows false colour render. (e) Degree of branching within fluid injection determines percentage of space defined as over-lit (>1000 lux of direct incident light). (f) Degree of branching within fluid injection determines average illuminance of space. (g) Degree of branching within fluid injection determines percentage of space defined as usefully lit (indicated here by a useful daylight illuminance fraction, or as the % of space lit at > 300 lux).

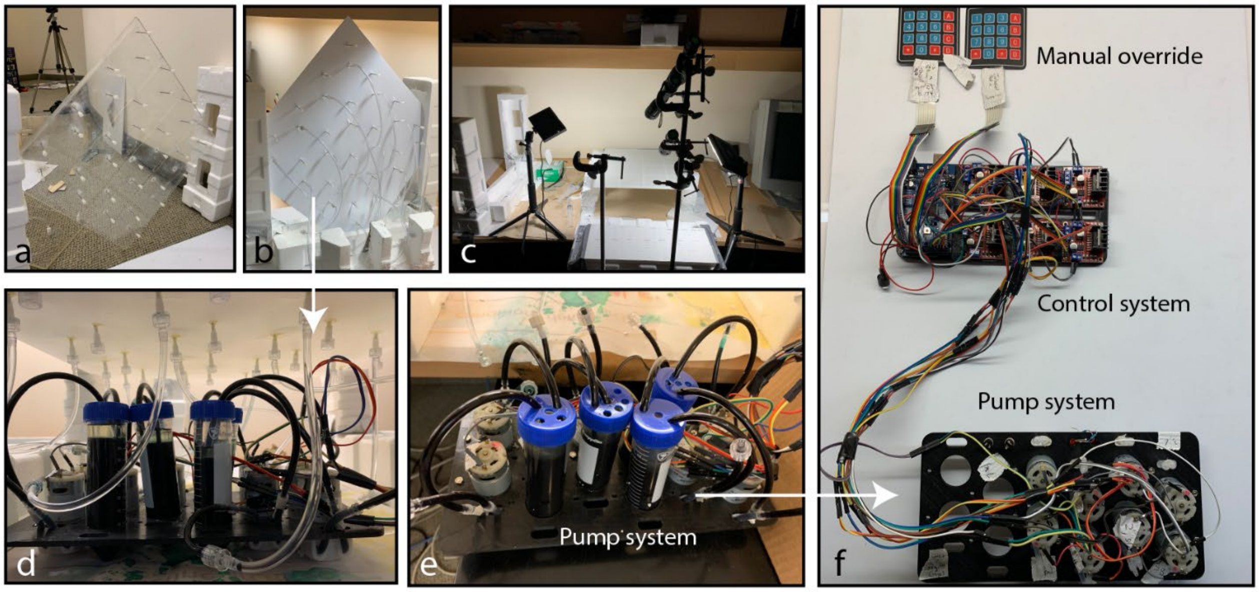

Supplementary Figure 13.

Photographs of experiments and devices. (a-b) Vertical multicell panels, rotated 45° for air bubble avoidance during preliminary oil injection. (c) Horizontal panel with targeted light intensity gradient setup. (d) Fluid pump system connected to panel. White backdrop added behind panels in (b-d) for better contrast during imaging. (e) Fluid panel system, disconnected from panel. (f) Electronics design, including fluid pump system, control system, and manual override system. Input light sensor wiring not pictured.

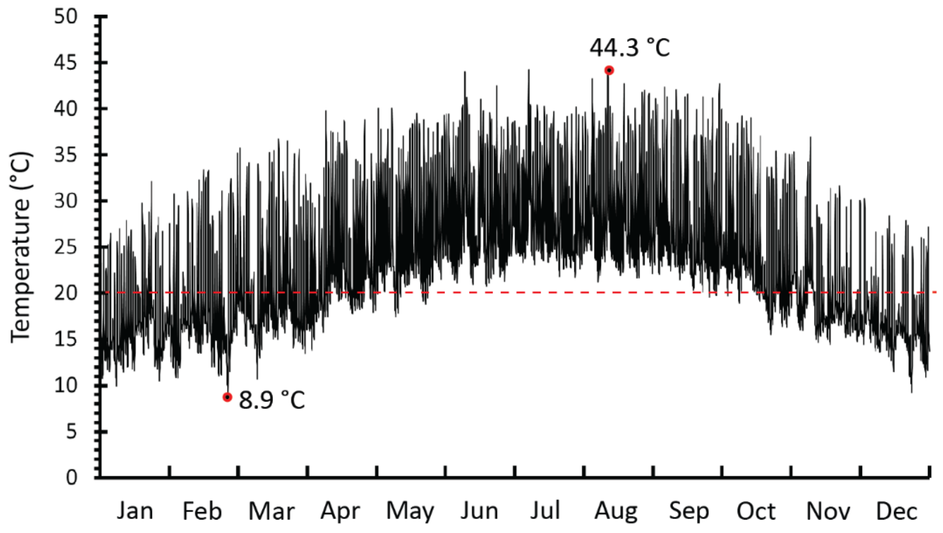

Supplementary Figure 14.

Simulated fluid temperature across the year, when modelled on inside of control double-glazed window in Toronto, Canada, as calculated using the conduction finite difference solution algorithm in EnergyPlus. The maximum fluid temperature was 44.3 °C on August 13th, and the minimum fluid temperature was 8.9 °C on February 23rd. The dashed red line denotes laboratory temperature of 20 °C. Using this data, we calculated the expected change in viscosity difference and the change in injection velocity required to appropriately compensate for these viscosity changes induced by temperature deviations.

Supplementary Table 1.

Required change in velocity to account for seasonal temperature fluctuations

**Condition**	**T (°C)**	**T - T_{lab} (°C)**	**n_g (Pa · s)**	**n_h (Pa · s)**	**Δn (Pa · s)**	**required V (mm/s) for constant λ_c**	**Required increase to V for constant λ_c**
Laboratory	20.0	0.0	0.00188	0.28723	0.28535	1.000 (arbitrary)	0%
Winter	8.9	-11.1	0.00281	0.66495	0.66214	0.431	**-57%**
Summer	44.3	+24.3	0.00110	0.06974	0.06864	4.158	**316%**

References

1 Ceylan, H. & Myers, G. E. Long-Time Solutions to Heat-Conduction Transients with Time-Dependent Inputs. *Journal of Heat Transfer-transactions of The Asme* **102**, 115-120 (1980).

2 U.S. Department of Energy. EnergyPlus Version 9.5.0 Engineering Reference. (2021).

3 Lane, L. B. Freezing Points of Glycerol and Its Aqueous Solutions. *Industrial & Engineering Chemistry* **17**, 924-924, doi:10.1021/ie50189a017 (1925).

4 Partridge, M. Calculate density and viscosity of glycerol/water mixtures. http://www.met.reading.ac.uk/~sws04cdw/viscosity_calc.html. Date accessed: May 1, 2021 (2018).

5 Volk, A. & Kähler, C. J. Density model for aqueous glycerol solutions. *Experiments in Fluids* **59**, 75, doi:10.1007/s00348-018-2527-y (2018).

6 Cheng, N.-S. Formula for the Viscosity of a Glycerol−Water Mixture. *Industrial & Engineering Chemistry Research* **47**, 3285-3288, doi:10.1021/ie071349z (2008).

7 Anton Paar. Viscosity of Engine Oil. https://wiki.anton-paar.com/ca-en/engine-oil/. Date accessed: May 1, 2021. (2021).
