# Supplementary material for: Decapod-inspired pigment modulation for active building facades
Source: Nat Commun. 2022 Jul 15;13:4120. doi: 10.1038/s41467-022-31527-6 (PMC9287369; doi:10.1038/s41467-022-31527-6)
Supplement: Supplementary file 3 — Description of Additional Supplementary Files [file 41467_2022_31527_MOESM3_ESM.pdf]

Description of Additional Supplementary Files

File Name: Supplementary Movie 1
Description: Comparison between biological and engineered intracellular pigment response in marine organisms and buildings. Left video shows a timelapse in reverse of fish melanophores responding to 200 μM of adrenaline. Right video shows viscous fingering of an aqueous fluidic pigment phase into a clear oil phase within a confined Hele-Shaw cell. The video on the left was extracted from 'Richard Wheeler. Fish Melanophores & Adrenaline. YouTube. Date of Upload: July 2, 2013. Date of Access: April 5, 2022' (https://youtu.be/hL0USeWjTHQ) and is licensed under a Creative Commons Attribution-Share Alike 3.0 Unported license: https://creativecommons.org/licenses/by-sa/3.0/deed.en.

File Name: Supplementary Movie 2
Description: Control over reversible pigment fluid morphology as function of flow rate.

File Name: Supplementary Movie 3
Description: Control over reversible pigment fluid morphology as function of viscosity ratio.

File Name: Supplementary Movie 4
Description: Stable, reversible, non-branching pigment fluid injection within a vertical cell.

File Name: Supplementary Movie 5
Description: Localized digital pigment fluid response to concentrated light intensity within 16-cell facade.

File Name: Supplementary Movie 6
Description: Digitally-programmed adaptive and differential pattern control within 16-cell facade.

File Name: Supplementary Movie 7
Description: Proportional and reversible pigmentary response to light intensity gradient across 16-cell facade.
